# Edema Toxin Impairs Anthracidal Phospholipase A2 Expression by Alveolar Macrophages

**DOI:** 10.1371/journal.ppat.0030187

**Published:** 2007-12-07

**Authors:** Benoit Raymond, Dominique Leduc, Lucas Ravaux, Ronan Le Goffic, Thomas Candela, Michel Raymondjean, Pierre Louis Goossens, Lhousseine Touqui

**Affiliations:** 1 Institut Pasteur, Unité de Défense Innée et Inflammation, Paris, France; 2 Unité Inserm, U.874, Paris, France; 3 UMR 7079, Physiologie et Physiopathologie Université Pierre et Marie Curie, CNRS, Paris, France; 4 Institut Pasteur, Unité des Toxines et Pathogénie Bactérienne, Paris, France; 5 CNRS, URA-2172, Paris, France; University of California Los Angeles, United States of America

## Abstract

Bacillus anthracis, the etiological agent of anthrax, is a spore-forming Gram-positive bacterium. Infection with this pathogen results in multisystem dysfunction and death. The pathogenicity of B. anthracis is due to the production of virulence factors, including edema toxin (ET). Recently, we established the protective role of type-IIA secreted phospholipase A2 (sPLA2-IIA) against B. anthracis. A component of innate immunity produced by alveolar macrophages (AMs), sPLA2-IIA is found in human and animal bronchoalveolar lavages at sufficient levels to kill B. anthracis. However, pulmonary anthrax is almost always fatal, suggesting the potential impairment of sPLA2-IIA synthesis and/or action by B. anthracis factors. We investigated the effect of purified ET and ET-deficient B. anthracis strains on sPLA2-IIA expression in primary guinea pig AMs. We report that ET inhibits sPLA2-IIA expression in AMs at the transcriptional level via a cAMP/protein kinase A–dependent process. Moreover, we show that live B. anthracis strains expressing functional ET inhibit sPLA2-IIA expression, whereas ET-deficient strains induced this expression. This stimulatory effect, mediated partly by the cell wall peptidoglycan, can be counterbalanced by ET. We conclude that B. anthracis down-regulates sPLA2-IIA expression in AMs through a process involving ET. Our study, therefore, describes a new molecular mechanism implemented by B. anthracis to escape innate host defense. These pioneering data will provide new molecular targets for future intervention against this deathly pathogen.

## Author Summary

All mammals are susceptible to infection by Bacillus anthracis, the etiological agent of anthrax. Infection can occur either accidentally or as a potential consequence of a terrorism threat. Pulmonary infection is the most life-threatening form of the disease, causing a near 100% mortality. Despite appropriate therapy, all forms of infection may progress to fatal systemic anthrax, characterized by sepsis and respiratory failure. Thus, it is important to understand the mechanisms of host defense against B. anthracis. We have previously shown that alveolar macrophages produce an enzyme involved in innate defense that can kill B. anthracis: the enzyme is known as secreted phospholipase A2-IIA (sPLA2-IIA). The alveolar macrophage is one of the first cell types to come in contact with B. anthracis. In this study, we show that live B. anthracis spores stimulate the synthesis of sPLA2-IIA, this stimulation being counterbalanced by the inhibitory effect of the edema toxin produced by germinated spores and bacilli. Our study suggests that inhibition of sPLA2-IIA synthesis by edema toxin is a mechanism by which B. anthracis can escape innate host defense. These pioneering data provide new molecular targets for future intervention against this deadly pathogen.

## Introduction

Bacillus anthracis, the etiological agent of anthrax, is a spore-forming Gram-positive bacterium [1]. Even though anthrax is primarily a disease of herbivores, all mammals are susceptible to B. anthracis infection. Human infection can occur via cutaneous, gastrointestinal, or respiratory routes, either accidentally or intentionally as a potential consequence of a bioweapon or a terrorism threat. Whatever the infection route used by this bacterium, spores are taken up by macrophages and/or dendritic cells, and subsequently migrate and germinate in the draining lymph nodes [2,3]. The infection then spreads through the whole organism, leading to respiratory failure and multiple hemorrhagic lesions. Despite appropriate therapy, all these forms of infection may progress to fatal systemic anthrax, which is characterized by shock-like symptoms, sepsis, and respiratory failure [4]. Pulmonary infection by B. anthracis has been shown to be the most life-threatening form of the disease, causing a near 100% mortality.

Innate immune response is the first line of host defense against invading pathogens. Type-IIA secreted phospholipase A2 (sPLA2-IIA) [5,6] is one of the major components involved in innate host defense against bacteria [7,8]. This enzyme belongs to a family of enzymes catalyzing the hydrolysis of phospholipids at the *sn-*2 position, leading to the generation of lysophospholipids and free fatty acids [5,6]. sPLA2-IIA is produced by several cell types, including guinea pig alveolar macrophages (AMs) [9], which play a central role in innate immunity and are the first line of defense against inhaled pathogens. These cells are the major pulmonary source of sPLA2-IIA in experimental models of acute lung injury [9,10]. Besides its ability to hydrolyze pulmonary surfactant phospholipids [11] and release arachidonic acid [12], sPLA2-IIA exhibits potent bactericidal activity, especially against Gram-positive bacteria [13–15]. The bactericidal activity is exhibited through a process involving rapid hydrolysis of bacterial membrane phospholipids [16,17]. This activity is the most significant biological property of sPLA2-IIA, being observed at much lower concentrations of this enzyme than for other properties. sPLA2-IIA is constitutively present in guinea pig airways at concentrations [11] above those required for killing B. anthracis [17]. We have also shown that isolated guinea pig AMs constitutively release enough sPLA2-IIA to kill B. anthracis [17]. sPLA2-IIA is highly bactericidal for B. anthracis, either in vitro or in vivo [17,18]. This anthracidal effect occurs both against germinated spores and bacilli. A sPLA2-IIA–dependent anthracidal activity was found in human bronchoalveolar lavage fluids (BALF) of patients with acute respiratory distress syndrome [17]. In a more recent study, we showed that transgenic mice expressing sPLA2-IIA are resistant to experimental infection with B. anthracis, in contrast to sPLA2-IIA^−/−^ mice [18]. Interestingly, treating sPLA2-IIA^−/−^ mice with recombinant sPLA2-IIA protected them against mortality caused by B. anthracis infection.

The exact mechanisms by which B. anthracis induces anthrax are not fully understood; however, it is clearly established that this bacterium spreads rapidly in the host at the early stages of infection without a detectable immune response [2,19,20]. This allows bacteria to replicate to very high numbers in the blood, ultimately leading to death of the host [4]. This is due to the ability of B. anthracis to subvert the host immune response [19,20] by the action of B. anthracis toxins. Indeed, B. anthracis secretes a binary A-B toxin composed of a single B transporter called protective antigen (PA) and two alternative A components, lethal factor (LF) or edema factor (EF) [1,19]. LF and EF act in pairs, with PA leading to lethal toxin (LT = PA + LF) and edema toxin (ET = PA + EF), respectively. PA serves as a transporter delivering LF and EF inside host cell cytosol where they act on specific molecular targets. EF is a calmodulin-activated adenyl-cyclase (AC) leading to a sustained increase in cAMP levels [21]. However, little is known about the genes targeted by ET and their implication in the pathogenesis of anthrax.

Here, we report that ET inhibits sPLA2-IIA expression in AMs and demonstrate that this inhibition occurs through the sequential accumulation of cAMP and stimulation of protein kinase A (PKA) activity. In the absence of ET, B. anthracis was able to induce sPLA2-IIA expression via a process at least partly involving the cell wall component, peptidoglycan (PG). ET also stopped this induction. This study is important because AMs are a major source of sPLA2-IIA, a critical component in host defense against *B. anthracis.* Inhibition of sPLA2-IIA expression in AMs by ET may represent an effective strategy for subverting pulmonary host immune response by B. anthracis.

## Results

### ET Impairs Lipopolysaccharide-Induced sPLA2-IIA Expression, but Not Interleukin 8 and Prostaglandin E2 Production, and Nuclear Factor κ B Translocation

**Figure 1:**
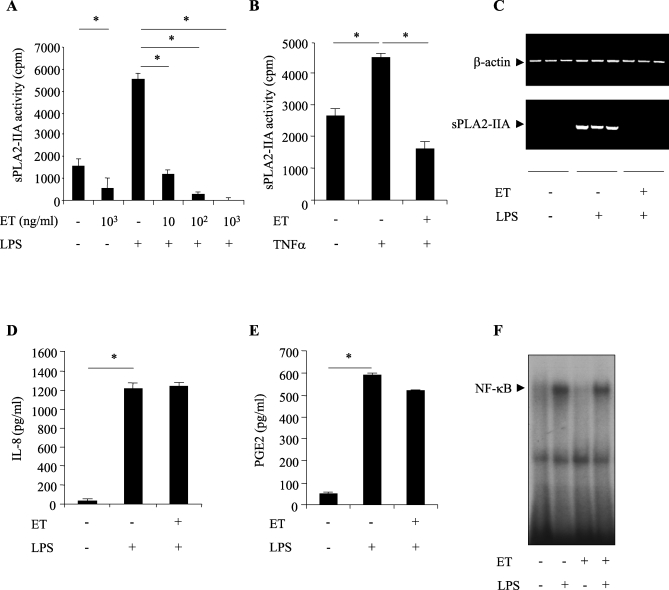
ET Impairs LPS-Induced sPLA2-IIA Expression, but Not IL-8, PGE2, and NF-κB Translocation (A) AMs were pretreated with ET (PA + EF) for 1 h before overnight stimulation with P. aeruginosa LPS (50 ng/ml). ET consisted of a fixed concentration of PA (1 μg/ml) and increasing concentrations of EF. (B) AMs were incubated with 1 μg/ml of ET (PA 1 μg/ml + EF 1 μg/ml), and were then stimulated overnight with TNFα (500 ng/ml). The sPLA2-IIA secretion levels were then assessed, as detailed in the Materials and Methods. (C–E) After 1-h pretreatment with 1 μg/ml of ET (PA 1 μg/ml + EF 1 μg/ml), followed by an overnight stimulation with LPS (50 ng/ml), sPLA2-IIA mRNA levels and IL-8 and PGE2 concentrations were measured in culture medium. (C) shows reverse-transcription PCR (RT-PCR) analysis of sPLA2-IIA mRNA expression of extracts from three independent experiments; (D) and (E) show IL-8 and PGE2 concentrations, respectively. (F) AMs were incubated with ET for 1 h and then were stimulated with LPS. After a 2-h incubation, NF-κB translocation was measured by EMSA. The data are the mean ± standard error of the mean (SEM), and are representative of four separate experiments. An asterisk (*) indicates *p* < 0.05.

AMs were preincubated with ET (PA + EF) 1 h before adding lipopolysaccharide (LPS) to analyze the effect of ET on sPLA2-IIA expression. ET stopped both basal and LPS-induced sPLA2-IIA secretion in a concentration-dependent manner (Figure 1A). No effect was observed when EF or PA was added separately to AMs (unpublished data). Inhibition of sPLA2-IIA secretion by ET was also observed when AMs were stimulated by tumor necrosis factor-α (TNFα) instead of LPS (Figure 1B). We showed that LPS induced a marked increase in sPLA2-IIA mRNA levels, and that the increase was subsequently abolished by the addition of ET (Figure 1C).

We next investigated the effect of ET on two other inflammatory mediators produced by AMs, interleukin 8 (IL-8) and prostaglandin E2 (PGE2). ET failed to interfere with LPS-induced IL-8 (Figure 1D) and PGE2 (Figure 1E) secretion. We also examined the effect of ET on nuclear factor κ B (NF-κB) translocation. ET had no effect on LPS-induced NF-κB translocation (Figure 1F), as assessed by electrophoretic mobility shift assay (EMSA). These results together indicated that ET inhibits sPLA2-IIA expression in AMs through a different signaling pathway from those inducing IL-8 and PGE2 secretion or NF-κB translocation.

### ET Impairs sPLA2-IIA Expression via a cAMP/PKA-Dependent Pathway

**Figure 2:**
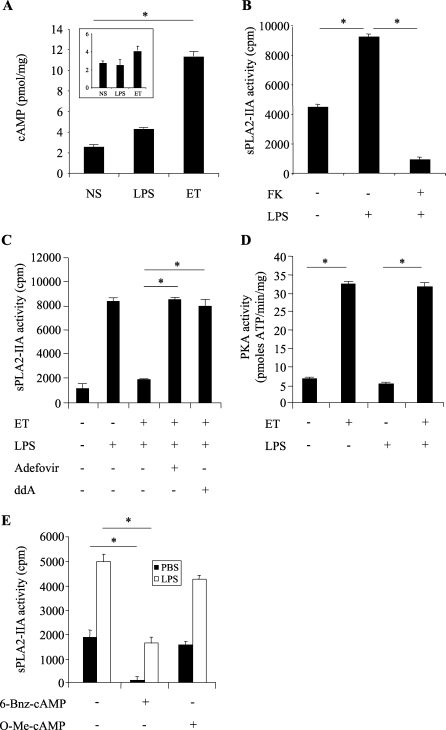
ET Impairs sPLA2-IIA Expression via a cAMP/PKA-Dependent-Pathway (A) AMs were treated with ET (1 μg/ml) and were then stimulated by LPS (50 ng/ml). After 30-min or 24-h (insert) incubation, cell lysates were collected and cAMP concentrations were measured by enzyme immunoassay (EIA) and expressed in picomoles per milligram of protein. NS, not stimulated. (B) AMs were treated with forskolin (FK; 20 μM) followed by LPS incubation. (C) Reversal of ET-mediated inhibition of sPLA2-IIA secretion by ET inhibitors. AMs were treated with adefovir (10 μM) for 5 h and incubated with ET (1 μg /ml) for 1 h. In a separate assay, ET (1 μg/ml) was treated with 100 μM ddA for 1 h and incubated with AMs for an additional 1 h. AMs were then stimulated with LPS (50 ng/ml) overnight, and supernatants were collected to measure sPLA2-IIA secretion. (D) After 2-h incubation, cell lysates were collected, and PKA activity was measured. (E) AMs were treated with 6-Bnz-cAMP or O-Me-cAMP (200 μM) for 1 h before overnight LPS stimulation. The data are the mean ± SEM and are representative of three separate experiments. An asterisk (*) indicates *p* <0.05.

Because ET exhibits a calmodulin-dependant AC, we examined its effect on intracellular cAMP levels in our cell system. A 30-min incubation of AMs with ET led to an increase in cAMP levels, whereas LPS had no effect (Figure 2A). The induction of cAMP accumulation by ET was transient; cAMP levels returned to near basal levels after AMs were incubated with ET for 24 h (Figure 2A, insert). In agreement, a cAMP-elevating agent, forskolin, significantly inhibited LPS-induced sPLA2-IIA secretion (Figure 2B). AC inhibitors (adefovir and ddA) reversed ET inhibition of LPS-induced sPLA2-IIA secretion (Figure 2C).

cAMP is known to activate protein kinases, such as PKA; thus, this kinase may be involved in the inhibition of sPLA2-IIA expression by ET. ET induced a marked and transient activation of PKA in AMs (Figure 2D). Indeed, this activation was observed 2 h after adding ET, and was undetectable 20 h later (unpublished data). To mimic the ET-induced PKA activation, we examined the effect of 6-Bnz-AMP, a specific agonist for PKA. 6-Bnz-AMP inhibited both basal and LPS-induced sPLA2-IIA expression (Figure 2E). By contrast, O-Me-cAMP, a specific agonist for the exchange protein directly activated by cAMP (Epac) [22], had no effect on sPLA2-IIA expression. Taken together, our results suggested that ET inhibits LPS-induced sPLA2-IIA expression in AMs via a cAMP/PKA-dependent process.

### Effect of ET on CREB Activation

**Figure 3:**
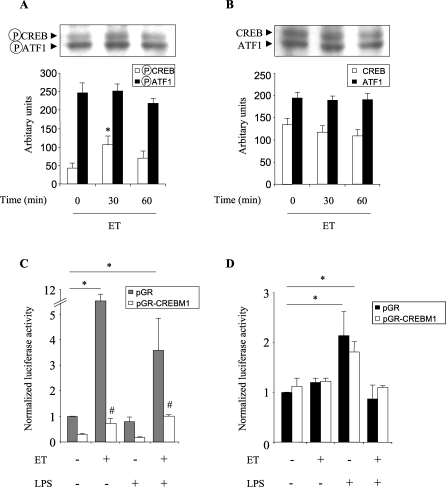
ET Induces CREB Activation AMs were incubated for 2 h with ET (1 μg/ml) and were then stimulated by LPS (50 ng/ml). After incubation for the time periods indicated, total cell proteins were extracted. (A and B) Western blot analyzes were then performed using phospho-CREB (ser 133) (A) or CREB (B) antibodies. The figure also shows the phosphorylation (A) and expression (B) levels of the activating transcription factor-1 (ATF-1). Corresponding quantifications were carried out using densitometry and were expressed as arbitrary units (representative of three separate experiments). (C) CHO cells were transfected with CREB ([CRE]4-Luc) reporter plasmid and/or dominant-negative CREB construct (pGR-CREBM1) or vehicle (pGR) for 24 h, pretreated for 1 h with ET (1 μg/ml) and stimulated with LPS (50 ng/ml) for an additional 24 h. (D) CHO cells were transfected with the sPLA2-IIA-Luc reporter plasmid and/or pGR-CREBM1. After 24-h transfection, cells were pretreated for 1 h with ET (1 μg/ml) and were stimulated with LPS (50 ng/ml) for an additional 24 h. Cells were then lysed, and the luciferase activity was measured and normalized with β-galactosidase activity. The data are the mean ± SEM and are representative of four separate experiments. An asterisk (*) indicates *p* < 0.05 ; a hash mark (#) indicates *p* < 0.05 compared to corresponding pGR controls.

Because PKA is known to phosphorylate cAMP-responsive element binding protein (CREB), we examined whether this transcription factor mediates the inhibition of sPLA2-IIA expression by ET. ET induced a time-dependent CREB phosphorylation (Figure 3A), but had no effect on the total level of CREB (Figure 3B) in AMs, as assessed by western blot analysis. We also investigated the effects of ET on CREB activation using Chinese hamster ovary (CHO) cells transfected with a CREB ([CRE]4-Luc) reporter plasmid construct. ET significantly increased the CREB luciferase activity (Figure 3C). However, LPS had no effect on this activity and failed to interfere with ET-induced CREB activation. This activation was prevented by cotransfecting cells with a dominant-negative CREB construct, pGR-CREBM1, as opposed to pGR (Figure 3C). Transfection of CHO cells with a sPLA2-IIA promoter luciferase construct demonstrated that ET inhibits LPS-induced sPLA2-IIA gene transcription activity (Figure 3D). Similar results were observed when LPS was replaced by IL-1β as the inducer of sPLA2-IIA expression (unpublished data). However, cotransfection of a dominant-negative CREB construct failed to reverse the inhibition of sPLA2-IIA gene transcription activity (Figure 3D), indicating that CREB does not mediate ET inhibition of sPLA2-IIA expression.

### Infection of AMs with Live B. anthracis Modulates sPLA2-IIA Expression

**Figure 4:**
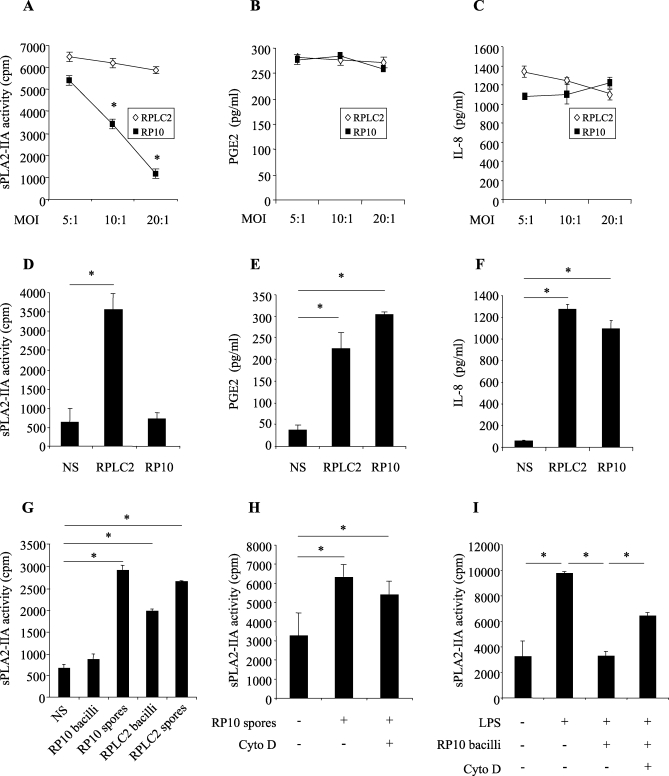
Infection of AMs with *B. anthracis* Inhibits sPLA2-IIA Secretion, but Not IL-8 or PGE2 Release (A–C) AMs were infected with B. anthracis (RP10 and RPLC2 strains) at the indicated MOI for 3 h in the absence of antibiotics. Cells were then washed and incubated overnight in RPMI 1640 supplemented with 3% FCS and antibiotics, in the presence or absence of LPS (50 ng/ml). Supernatants were collected, and sPLA2-IIA secretion and PGE2 and IL-8 concentrations were measured. Effects of bacilli on sPLA2-IIA secretion (A) and PGE2 (B) and IL-8 (C) production in LPS-stimulated AMs. (D and E) Effect of bacilli (MOI 20:1) on sPLA2-IIA secretion (D), and PGE2 (E) and IL-8 (F) production in unstimulated AMs. NS, not stimulated. (G) Effect of spores and bacilli (MOI 20:1) on sPLA2-IIA secretion (G) in unstimulated AMs. (H and I) Effect of Cyto D on the modulation of sPLA2-IIA expression by RP10 spores (H) and bacilli (I). AMs were incubated with 3 μM Cyto D for 30 min before addition of bacteria (MOI 20:1). AMs were washed 3 h later and incubated in the presence (I) or absence (H) of LPS. The data are the mean ± SEM and are representative of four separate experiments. An asterisk (*) indicates *p* < 0.05.

Using a more pathophysiological approach, we examined whether infecting AMs with B. anthracis bacilli modulates sPLA2-IIA expression and whether ET participates in this modulation. AMs were incubated in an antibiotic-free culture medium for 3 h with either RP10 or RPLC2 bacilli; RP10 produces functional and RPLC2 produces inactive ET. After removing bacilli not having undergone phagocytosis, AMs were stimulated overnight with LPS in culture medium supplemented with antibiotics. The RP10 strain inhibited LPS-stimulated sPLA2-IIA secretion, whereas the RPLC2 strain had no effect (Figure 4A). Inhibition by the RP10 strain occurred in a multiplicity of infection (MOI)-dependent manner and was selective for sPLA2-IIA. Indeed, this strain failed to inhibit LPS-induced PGE2 (Figure 4B) and IL-8 (Figure 4C) production. These findings demonstrated that in LPS-stimulated AMs, B. anthracis strains producing functionally active ET down-regulated sPLA2-IIA expression.

We next examined the effect of B. anthracis on sPLA2-IIA expression in unstimulated AMs. RPLC2 bacilli induced sPLA2-IIA expression (Figure 4D), and PGE2 (Figure 4E) and IL-8 (Figure 4F) secretion. The RP10 bacilli strain induced PGE2 and IL-8 secretion, but had no effect on sPLA2-IIA expression (Figure 4D–4F). Interestingly, RP10 and RPLC2 spores induced sPLA2-IIA expression, even after 3 h of infection (Figure 4G). These findings indicate that in the sporular state, RP10 and RPCL2 strains induce sPLA2-IIA expression. However, in the bacilli state, the RPCL2 strain (devoid of ET) induced sPLA2-IIA expression, whereas the RP10 strain (producing ET) exerted an inhibitory effect. AMs were incubated with cytochalasin D (Cyto D) before adding the RP10 strain, to examine the impact of B. anthracis phagocytosis on sPLA2-IIA expression. Cyto D reduced the inhibitory effect of bacilli on sPLA2-IIA expression, but failed to interfere with the stimulatory effect of spores (Figure 4H and 4I). This suggests that both extracellular and intracellular bacilli are involved in inhibiting sPLA2-IIA expression, whereas extracellular spores seem to play a more important role in inducing this enzyme.

### B. anthracis Peptidoglycan Induces sPLA2-IIA Expression That Is Inhibited by ET

**Figure 5:**
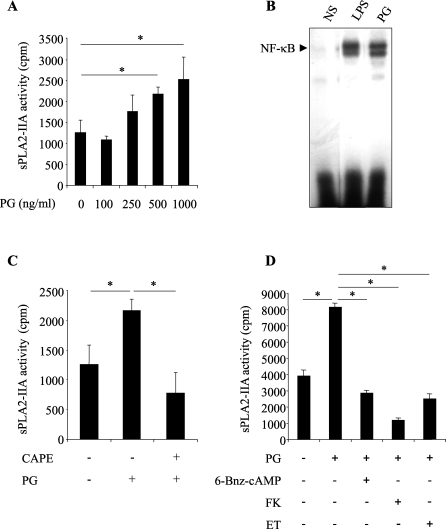
B. anthracis PG Induces sPLA2-IIA Expression (A) After AMs were stimulated overnight with the indicated concentrations of PG, sPLA2-IIA secretion was examined. (B) After a 1-h incubation with PG (500 ng/ml) or LPS (50 ng/ml), the nuclear extracts were obtained and EMSA was performed. NS, not stimulated. (C) AMs were incubated for 1 h with CAPE (10 μM), followed by an overnight stimulation with PG (500 ng/ml). (D) AMs were pretreated with 6-Bnz-cAMP (200 μM), FK (20 μM), or ET (1 μg/ml) for 1 h before overnight stimulation with B. anthracis PG (2 μg/ml). Then, the supernatants were collected to measure sPLA2-IIA secretion. The data are the mean ± SEM and are representative of four experiments. An asterisk (*) indicates *p* < 0.05.

As RPLC2 strain induces sPLA2-IIA expression in AMs, we searched for which B. anthracis component was involved in this induction. PG purified from B. anthracis stimulated sPLA2-IIA expression (Figure 5A). PG, as well as LPS, induced NF-κB translocation, as assessed by EMSA (Figure 5B). PG-induced sPLA2-IIA expression was abolished if AMs were pretreated with the NF-κB inhibitor CAPE (Figure 5C). Interestingly, pretreating AMs with ET stopped sPLA2-IIA expression induced by B. anthracis PG (Figure 5D). PG-induced sPLA2-IIA expression was also inhibited by the cAMP-elevating agent, forskolin, and the PKA agonist, 6-Bnz-cAMP (Figure 5E).

## Discussion

In this study, we investigated the effect of B. anthracis, the causative agent of anthrax [1–4], on the expression of sPLA2-IIA, an important component of host defense against invading bacteria. This enzyme is bactericidal in vitro or in vivo, and is especially active against Gram-positive bacteria, including B. anthracis [7,8,17,18]. sPLA2-IIA is produced by AMs and found in human and animal BALF at sufficient levels to kill B. anthracis [17]; these findings are consistent with the enzyme having a role in host defense against pulmonary anthrax. However, despite the ability of lungs to produce sPLA2-IIA, the pulmonary form of anthrax has been shown to be fatal, causing almost 100% mortality [1–4]. This led us to postulate that B. anthracis may inhibit sPLA2-IIA synthesis by AMs, subvert host pulmonary defense, and allow this pathogen to spread extensively in the host.

We show here that ET inhibits sPLA2-IIA secretion by AMs, interfering with its expression at the transcriptional level. Inhibiting sPLA2-IIA secretion may decrease the capacity of AMs to kill B. anthracis bacilli and germinated spores. Indeed, AM activity against B. anthracis has been shown to be at least partly associated with sPLA2-IIA, as it was reduced by an sPLA2-IIA inhibitor [17]. This inhibition was observed whatever the stimuli used (LPS, TNFα , IL1β, or PG). We analyzed the signaling pathways by which ET down-regulates sPLA2-IIA expression; our analysis suggested that this inhibition occurs via a process involving cAMP accumulation. Our studies showed that this accumulation was transient, reaching near basal values within 24 h. This contrasts with previous studies reporting that cAMP accumulation was elevated for 48 h or more after ET incubation with NIH/3T3 fibroblasts and RAW 267 macrophages [23]. Thus, it is likely that the duration and amplitude of cAMP accumulation induced by ET may vary with the cell type considered. Because cAMP activates several kinases, we examined whether PKA and Epac, two cAMP-dependent kinases, were involved in this process. PKA but not Epac, appeared to mediate ET-induced inhibition of sPLA2-IIA expression. Our results also suggested that elevating intracellular cAMP concentrations (either by ET or 6-Bnz-cAMP) interfered with basal and LPS-induced sPLA2-IIA expression by different mechanisms. The inhibition of induced expression appeared to occur through a process that interferes, at least partly, with the sPLA2-IIA promoter, whereas the inhibition of basal expression appeared to be independent of the sPLA2-IIA transcription.

PKA phosphorylates proteins, such as CREB, that are involved in regulating gene expression in mammalian cells [24]. This factor can modulate, either positively or negatively, gene expression in several cell-activation processes [24,25]. Although ET induces CREB activation, this transcription factor does not mediate the inhibition of sPLA2-IIA expression by ET. However, it is likely that CREB activation by ET could modulate the expression of other genes involved in host defense, which remain to be identified. We next investigated whether ET inhibits sPLA2-IIA expression by interfering with the activation of NF-κB, known to be critical in inducing sPLA2-IIA expression [26]. ET had no effect on stimulated NF-κB translocation in AMs. Also, ET had no effect on the secretion of IL-8, whose expression is controlled by NF-κB. However, we cannot exclude that ET may interfere with stimulating cofactors involved in NF-κB coactivation at the sPLA2-IIA promoter level. Studies in progress in our laboratory showed that trichostatin A, an inhibitor of histone deacetylase (HDAC) activity [27], significantly decreased sPLA2-IIA expression in LPS-stimulated AMs. Because HDAC activity is altered by a PKA-dependent phosphorylation [28], it is likely that HDAC may play a role in the inhibition of sPLA2-IIA expression by ET. Further studies are required to verify this hypothesis.

**Figure 6:**
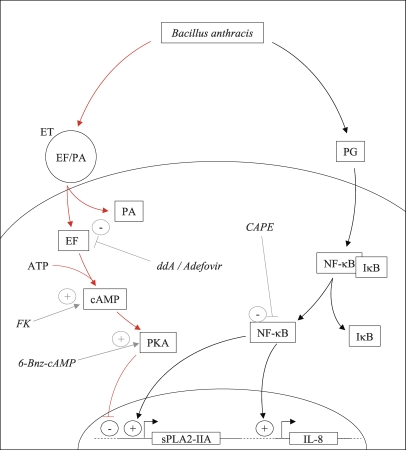
A Schematic Model for the Modulation of sPLA2-IIA Expression in AMs by B. anthracis At the earlier stages of infection, PG constitutively present in B. anthracis cell walls induces sPLA2-IIA and IL-8 expression via an NF-κB–dependent process. After being taken up by AMs, B. anthracis spores undergo germination, which subsequently leads to bacilli formation and ET production. ET down-regulates sPLA2-IIA expression through sequential cAMP accumulation and PKA activation. The mechanisms by which PKA activation leads to down-regulation of sPLA2-IIA expression remains to be elucidated. As sPLA2-IIA is involved in pulmonary host defense against B. anthracis, its repression may lead to the proliferation of this bacterium in lung tissues. The black arrows represent the stimulatory pathway involving spores, and the red arrows correspond to the inhibitory pathway involving bacilli. ET appears to inhibit basal expression and induce sPLA2-IIA expression by various mechanisms (for more detail, see Discussion). IκB, inhibitor of κ B.

In a more physiological approach, we investigated whether ET modulates sPLA2-IIA expression during infection of AMs with live *B. anthracis.* This bacterium inhibits LPS-induced sPLA2-IIA expression via ET. Indeed, RPLC2, the bacterial mutant with inactive ET, had no effect on this induction, whereas the RP10 strain expressing functional ET abolished LPS-induced sPLA2-IIA expression. Incubating RPLC2 bacilli, which produce inactive ET, with unstimulated AMs induced sPLA2-IIA expression. This suggested the existence of bacterial component(s) that are able to induce sPLA2-IIA synthesis, and that their actions are masked by the ET inhibitory effect produced by RP10 bacilli. Our findings showed that the cell wall PG purified from B. anthracis induces sPLA2-IIA expression via a process involving NF-κB activation. It is still not clear whether PG-induced sPLA2-IIA expression occurs via an activation of TLR2 or Nod, two PG recognition proteins [29]. A recent study has reported that Nod may be involved in cell activation by B. anthracis spores [30]. We cannot exclude, however, that other bacterial components present in the cell wall or released by B. anthracis may also be involved in inducing sPLA2-IIA expression. Interestingly, ET suppressed PG-induced sPLA2-IIA expression, confirming the relevance of our studies, and showing that ET also suppresses the sPLA2-IIA expression induced by B. anthracis itself. Therefore, during host infection, B. anthracis may modulate sPLA2-IIA expression, either positively or negatively, depending on the status of ET synthesis in the bacterium (Figure 6).

Mammalian pulmonary infection with B. anthracis is initiated by the inhalation of spores, the cell walls of which contain PG. Infecting spores therefore induce sPLA2-IIA expression in the earlier stages of infection. This is consistent with previous studies, which have reported that B. anthracis spores stimulate cytokine production in various cells [31–33]. The susceptibility of inhaled spores to the bactericidal activity of sPLA2-IIA present in airways is dependent on their germination velocity; this is because sPLA2-IIA only kills germinated spores and bacilli [17]. Previous in vivo studies [3] have shown that germination occurred rapidly upon entry into the lung (35–60 min), and that the spores were mostly found inside the AM. This was followed by a rapid onset (<3 h) of expression of genes encoding virulence factors, such as LF, PA, and EF [34]. Elimination of inhaled B. anthracis by the host would thus depend on the balance between sPLA2-IIA levels in the airways and bacterial load. If the balance favors sPLA2-IIA, germinated spores and bacilli would be killed quickly. Our previous studies have shown that the constitutive (basal) levels of sPLA2-IIA present in guinea pig airways [11] are greater than those required for killing B. anthracis [17], and that these levels were greater in inflamed lungs [11]. sPLA2-IIA was also found in BALF of patients with lung inflammatory diseases (ARDS) at sufficient levels to exert this anthracidal effect. However, it is still unknown whether BALF of healthy subjects contains functionally significant amounts of sPLA2-IIA, and whether this enzyme would be available soon after the invasion of inhaled spores. It is also possible that some germinated bacteria may escape killing by sPLA2-IIA. Therefore, bacilli derived from geminated spores rapidly produce ET, which may in turn inhibit sPLA2-IIA expression and shift the balance in favor of bacteria. In vivo studies, in which guinea pigs are infected with inhaled B. anthracis spores, are required to investigate this possibility.

Our previous studies clearly established a role for sPLA2-IIA in host defense against B. anthracis; however, it is still unknown whether this enzyme participates in killing germinated spores previously ingested by AMs. sPLA2-IIA is involved in the killing of Staphylococcus aureus ingested by neutrophils [35]. Indeed, added sPLA2-IIA binds to S. aureus before its internalization by neutrophils and participates in the cytotoxicity of these cells towards ingested bacteria. Therefore, we suggest that sPLA2-IIA is involved in killing B. anthracis ingested by AMs.

In conclusion, we report here that B. anthracis represses the expression of sPLA2-IIA, a major component in innate host response with anthracidal properties, in AMs. This inhibition occurs through a process involving ET-mediated cAMP accumulation and PKA activation, and represents a novel mechanism for evading the innate immune response of the host. Other bacteria (for example Bordetella pertussis or Yersinia pestis) [19] are known to produce toxins with AC activity; thus, we can speculate that the inhibition of sPLA2-IIA expression in AMs may be a more general process occurring during bacterial infection. Therefore, using pharmacological approaches to inhibit ACs of invading bacteria may represent a therapeutic strategy for treating not only pulmonary anthrax, but also other bacterial pulmonary infections.

## Materials and Methods

### Animals and reagents.

Male Hartley guinea pigs were purchased from Charles River Laboratories. RPMI 1640 cell culture medium was purchased from Invitrogen, and fetal calf serum (FCS) from Hyclone. Caffeic acid phenethyl ester (CAPE), and cytochalasin D (Cyto D) were purchased from Biomol. LPS from Pseudomonas aeruginosa and 2′, 5′-dideoxyadenosine 3′-triphosphate (ddA) were purchased from Sigma Aldrich. N^6^-Benzoyladenosine-3′, 5′-cyclic monophosphate (6-Bnz-cAMP) and 8-(p-Chlorophenylthio)-2′-O-methyl-adenosine-3′, 5′-cyclic monophosphate (O-Me-cAMP) were purchased from Biolog. CREB and phospho-CREB antibodies were obtained from Cell Signaling Technology. EF, PA, and PG from B. anthracis were produced and purified as described previously [36]. BIS-POM-PMEA (adefovir) was provided by Dr. W. J. Tang (University of Chicago, Chicago, Illinois).

### B. anthracis strains.

The following isogenic B. anthracis strains were studied: (1) the single mutant RP10 Δ*lef* producing only PA-EF and (2) the double-mutant RPLC2 on *lef* and *cya* genes producing PA-LF and PA-EF, respectively, without enzymatic functions [37].

### Treatment of AMs with bacterial toxins and drugs.

Guinea pig bronchoalveolar lavages (BAL) were performed with PBS, and AMs were isolated, as previously described [9]. AMs were then adjusted at 2.10^6^ cells/ml in RPMI 1640 with 3% FCS and 1% of antibiotic, and were pretreated with ET (PA + EF), 6-Bnz-cAMP, O-Me-cAMP, or TSA 1 h before incubation with LPS, PG, or TNFα. In certain experiments, AMs were pretreated 5 h with adefovir before incubation with ET. In other experiments, ET was preincubated with ddA for 1 h before being added to AMs. These reagents were used at the concentrations indicated in the figures. Subsequent analyzes were performed as detailed below.

### Infection of AMs.

Cells were infected with B. anthracis bacilli or spores for 3 h at various MOI values. Cells were then washed twice and incubated overnight in RPMI 1640 supplemented with 3% FCS and 2.5 μg/ml gentamicin in the presence or absence of LPS. In certain experiments, AMs were pretreated with Cyto D for 30 min before adding bacteria. At the end of the incubation, media were harvested and centrifuged. The resulting supernatants were collected and stored at −20 °C for subsequent analyzes.

### RNA extraction and reverse-transcription PCR analysis.

Cells were grown on a cell culture plate and total RNA was extracted using an RNeasy kit (Qiagen). DNase treatment was performed using 2 μg of extracted RNA, 1 μl of DNase I (Amersham Biosciences), and 0.5 μl of RNasin (Promega) in a total volume of 20 μl in the manufacturer's buffer. cDNA were obtained by incubating RNA with 1 mM dNTP (Eurobio), 1.5 μl of hexamers as primers, 20 units of RNasin (Promega), and 300 units of Moloney murine leukemia virus reverse transcriptase RNase H minus (Promega) in a total volume of 50 μl of the manufacturer's buffer; the incubation was for 1 h at 42 °C and was followed by a 10-min incubation at 70 °C. PCR was performed using specific primers (Proligo) for guinea pig sPLA2-IIA (sense, 5′-ACA AGT TAT GGC GCC TAT GG-3′; antisense, 5′-GCC CAG TGT AGC TGT GAA GC-3′). As an internal control, we used primers for the detection of guinea pig β-actin (sense, 5′-AAA CTG GAA CGG TGA AGG TG-3′; antisense, 5′-TCA AGT TGG GGG ACA AAA AG-3′). Amplifications were performed in a Peltier thermal cycler (MJ Research) using Q-BioTaq polymerase (Qbiogene). For the detection of sPLA2-IIA, PCR thermo-cycling included 30 cycles of denaturation at 95 °C for 45 s and annealing at 60 °C for 45 s.

### Nuclear protein extraction and electrophoretic mobility shift assays.

Nuclear proteins were extracted from 2.10^6^ AMs, as previously described [38]. The NF-κB double-stranded oligonucleotides (Santa Cruz Biotechnology) corresponded to an NF-κB binding site consensus sequence of 5′-AGT TGA GGG GAC TTTT CCC AGG C-3′. The overhanging ends were γ-^32^P–labeled with T4 polynucleotide kinase (Biolabs). Protein concentrations were determined using a Nanodrop spectrophotometer (Nyxor Biotech). Binding reactions were performed in a total volume of 20 μl for 20 min at room temperature, by adding 5 μg of nuclear extract, 10 μl of 2× binding buffer (40 mM HEPES [pH 7], 140 mM KCl, 4 mM DTT, 0.02% Nonidet P-40, 8% Ficoll, 200 μg/ml BSA, 1 μg of poly(dI:dC)), and 1 μl of labeled probe. The reaction mixtures were separated on a 5% polyacrylamide gel in 0.5% Tris/borate/EDTA buffer at 150 V for 2 h. Gels were dried and exposed for 2 to 12 h. We have previously shown, using supershift analysis, that antibodies directed against NF-κB's p50 and p65 subunits displaced the NF-κB band in LPS-stimulated AMs; this confirmed that the observed complexes belong to the NF-κB family [39].

### Protein extraction and western blot analyzes.

Proteins from AMs were extracted in lysis buffer (10 mM Tris-HCl, 10 mM NaCl, 3 mM EDTA, 100 μM leupeptin, 100 mM aprotinin, 1 mM soybean trypsin inhibitor, 5 mM NEM, 1 mM PMSF, 5 mM benzamidine, and 1% Triton W-100 [pH 7.4]) and were run on a gel under reducing conditions. Semidry transferred proteins were applied to polyvinylidene difluoride membranes. Nonspecific binding sites were blocked overnight with 5% BSA in 20 mM Tris-HCl (pH 7.6), 140 mM NaCl, and 0.1% Tween 20. Blots were probed for 1 h with rabbit polyclonal anti-human phospho-CREB (ser 133) or CREB antibodies (1/2,000 dilution). These antibodies also recognize activating transcription factor-1 (ATF-1), which belongs to the CREB family. After washing, the immunoreactive bands were visualized using a peroxidase-conjugated goat anti-rabbit immunoglobulin G (IgG) (1/10,000 dilution) antibody and an ECL Plus Western Blotting Detecting System (Amersham Biosciences). Quantifications were carried out using the Image J software and were expressed as arbitrary units.

### cAMP, PGE2, and IL-8 enzyme immunoassays.

cAMP concentrations were measured in disrupted cells using a specific enzyme immunoassay kit purchased from Cayman Chemical Co; the concentrations were measured after incubating AMs with LPS and/or ET for 30 min or 24 h. Protein concentrations were measured in cell lysates using a kit from Pearce, and then the concentrations of cAMP were expressed in picomoles per milligram of protein. IL-8 and PGE2 concentrations were measured in culture medium after 24 h incubation of AMs with LPS and/or ET; these concentrations were measured using a specific PGE2 enzyme immunoassay (Cayman Chemical Co) and human IL-8 Kit DuoSet ELISA (R&D Systems), which cross-reacts with guinea pig IL-8 [40].

### PKA and sPLA2-IIA activity assays.

AMs were incubated with LPS and/or ET for 2 h. The PKA activity was then measured in disrupted cells using a specific enzyme immunoassay kit purchased from Promega. sPLA2-IIA activity was measured in culture medium using [^3^H]-oleic acid-labeled membranes of Escherichia coli, following a modification [41] of the method by Franson et al. [42].

### Plasmid constructions and transfection.

Mutated constructs [−488; +46]-sPLA2-Luc were prepared, as described previously [43]. CHO cells were seeded on dishes cultured in HAM F12, supplemented with 10% (v/v) FCS (Gibco BRL), 4 mM glutamine, 100 U/ml penicillin, and 100 mg/ml streptomycin. CHO cells were seeded in 24-well plates at a concentration of 2.10^4^ cells per plate at 70% confluence, 24 h before transfection. Transfections with mutated constructs [−488; +46]-sPLA2-Luc, CREB, and DN CREB were performed using 0.75 ml of LIPOFECTAMINE Plus (Invitrogen), 0.4 mg of reporter DNA, as indicated in Figure 4, and 0.1 mg of pCMV-β-galactosidase per well. The cells were incubated with HAM-F12 medium 3 h after adding the DNA, and incubation was continued for 24 h. CHO cells were incubated with PA (1 μg/ml) and EF (500 ng/ml) for 1 h. LPS (1 μg/ml) was then added, and incubation was continued for an additional 24 h. Luciferase activity was measured using a luciferase reporter assay kit, with signal detection for 12 s by a luminometer (Berthold), and was normalized by dividing the relative light units by β-galactosidase activity [43]. The degree of induction was calculated relative to the control.

### Control of cell viability.

Cell viability was checked by the trypan blue dye exclusion test. Cell lysis was controlled by measuring the release of lactate dehydrogenase (LDH) activity using a commercial kit from Boehringer. No cell mortality was observed in all the experiments presented in this study.

### Statistical analysis.

Data are expressed as the mean ± standard error of the mean (S.E.M.) of at least three separate experiments, and statistical analyzes were performed using the unpaired Student *t*-test.

## Abbreviations

AC: adenyl-cyclase
AM: alveolar macrophage
BALF: bronchoalveolar lavage fluids
CHO: Chinese hamster ovary
CREB: cAMP-responsive element binding protein
Cyto D: cytochalasin D
EF: edema factor
EMSA: electrophoretic mobility shift assay
IL: interleukin
LF: lethal factor
LPS: lipopolysaccharide
MOI: multiplicity of infection
NF-κB: nuclear factor κ B
PA: protective antigen
PG: peptidoglycan
PGE2: prostaglandin E2
PKA: protein kinase A
SEM: standard error of the mean
sPLA2-IIA: type-IIA secreted phospholipase A2
TNFα: tumor necrosis factor-α
